# Patient satisfaction with the perioperative surgical services and associated factors at a University Referral and Teaching Hospital, 2014: a cross-sectional study

**DOI:** 10.11604/pamj.2017.27.176.10671

**Published:** 2017-07-05

**Authors:** Endale Gebreegziabher Gebremedhn, Girmay Fitiwi Lemma

**Affiliations:** 1 Department of Anaesthesia, School of Medicine, Gondar College of Medicine and Health Sciences, the University of Gondar, Gondar, Ethiopia; 2 Department of Anaesthesia, School of Medicine, Gondar College of Medicine and Health Sciences, the University of Gondar, Gondar, Ethiopia

**Keywords:** Patient satisfaction, perioperative surgical services, contributing factors

## Abstract

**Introduction:**

Globally, increasing consideration has been given to the assessment of patient satisfaction as a method of monitor of the quality of health care provision in the health institutions. Perioperative patient satisfaction has been contemplated to be related with the level of postoperative pain intensity, patients’ expectation of the outcome, patient health provider relationship, inpatient services, hospital facilities, access to care, waiting time, cost and helpfulness of treatments received. The study aimed to assess the level of patient satisfaction with perioperative surgical services and associated factors.

**Methods:**

Hospital based quantitative cross-sectional study was conducted in University of Gondar teaching hospital from April1-30, 2014. Structured Amharic version questionnaire and checklist used for data collection. All patients who operated upon during the study period were included. Both bivariate and multivariate logistic regression model used to identify the variables which had association with the dependent variable. P-values < 0.05 were considered statistically significant.

**Results:**

Two hundred and seventy eight patients underwent surgery during the study period. Nine patients were excluded due to refusal to participate in the study. A total of 269 out of 278 patients were included in the study with a response rate of 96.8%. The overall level of patient satisfaction with perioperative surgical services was 98.1%. The variables that had association with the outcome variable from the multivariate analysis were patient admission status (AOR=0.073, CI=0.007-0.765, P=0.029), information about the disease and operation (AOR=0.010, CI=0.001-0.140, P=0.001) and operation theatre staff attention to the patients complains (AOR=0.028, CI=0.002-0.390, P=0.008) respectively.

**Conclusion:**

The level of patient satisfaction with perioperative surgical services was high compared with previous studies conducted in the country and other countries in the world. Health professionals need to give emphasis for information on care provision processes, patients' health progress and patients' complaints.

## Introduction

Patient satisfaction is one of the main indicators of patient experience about health care services and quality of care provided [1, 2]. Patient satisfaction is also considered as one of the required outcomes of health care and it is directly related with utilization of health services. Assessing patients what they think about the care and treatment they have received is an important process towards improvement of the quality of care, to ensure whether the local health services are meeting patients’ needs and identify possible barriers for the delivery of the services [3]. Patient satisfaction with perioperative service is another complex area where satisfaction can be affected by many factors. Patients may choose a different health institution and surgeon depending on their anticipation and overall satisfaction with the care provided [4]. Preoperative anxiety, limited functional status, and postoperative pain control are some of the important aspects in the management of surgical patients and are interrelated to a successful recovery and patient satisfaction [5]. The perioperative area is a distinctive environment that includes many challenging conditions: multifaceted clinical care performed by teams; high cost, use of sophisticated technologies and a large variety of supplies, instruments, and implants that are difficult to manage. These conditions can create an environment of massive complexity and, unfortunately, are a source of a significant percentage of patient safety related adverse events. Some of the kinds of errors that can occur during surgery are patient misidentification, surgical site misidentification, and medication errors and omissions [6–8]. There is increasing appreciation that clinically based measures of surgical outcome must be accepted with patients' awareness of their post-treatment situation and patient satisfaction research is becoming an independent growth indicator where emphasis is given for patient feedback on quality of care received [9].

There are many aspects of patient satisfaction with treatment such as overall satisfaction, interpersonal factors, competence of professionals, patient ranking of the result, materials, continuity of care, accessibility for facilities, information about procedure and treatment, system of service, and costs for treatment [10]. Patient satisfaction could be affected by patients’ expectations, overall health status, psychological factors and nature of treatment of provided [11, 12]. Preoperative anticipations of patients were the main predictors of patient experiences, dissatisfaction and mood disorder after operation [13–15]. Similarly, a study conducted in Norway showed that adequate information about their health status, treatment options, and relation with nursing and medical staffs were the major determinants of patient satisfaction [16]. On the other hand, perioperative discomforts were the main factors that affected patient satisfaction negatively [17]. On the contrary, quality of information did not have an effect on patient satisfaction but admission processes, information provision, nursing care, physician and nurse interactions affected surgical in-patient satisfaction [18]. Another study showed that postoperative pain, waiting time for surgery and patient changing room conditions were the most important factors influencing patient satisfaction [19]. Older inpatients were more satisfied whereas younger outpatients were more satisfied. The most influential factors on inpatient satisfaction were information at admission, knowing what type of professional was dealing about the patient any time and informed consent. And for outpatients, informed consent and information about home care after discharge were the most influential factors [20]. A study done in Brigham on patient satisfaction in a tertiary teaching hospital preoperative clinic services showed that assessment of patient load and clinic service delivery system led to alterations in the service processes that resulted in continued high clinical effectiveness, reduced waiting time, and improved patient satisfaction [21]. Younger patients and patients who had postoperative complication after breast surgery were less satisfied [22]. Surgical patients were moderately satisfied with nursing care in orthopedic wards and were highly satisfied with nurses’ manner in going about their work but were less satisfied with the amount of time nurses spent with them [23].

A prospective cohort study done on 4709 patients about patient’s satisfaction after joint arthroplasty was significantly affected by meeting of preoperative patients’ expectations, symptomatic pain management after surgery and the overall hospital experience [24]. A study conducted in Cyrus showed that patients were more satisfied with the technical aspect of nursing care but were less satisfied with the provision of information and hospitalisation especially with food and resting time [25]. A study conducted on determinants of patient satisfaction in surgical ward at a University Hospital in Saudi Arabia showed that explanation of a responsible physician for operation in the emergency department, physician’s reception in the clinic, surgical team reception in the ward, response of the team about the patient's questions and safety level in the hospital affected patient satisfaction positively. Whereas waiting time in the emergency, waiting time in the clinic, the response of consulting doctors from other departments, explanation of the surgical team about the life style after operation and the quality of food in the hospital affected patient satisfaction negatively [26]. Similarly, a study conducted in Scotland on satisfaction of patients after day surgery revealed that waiting times between admission, operation, and discharge , and postoperative pain affected satisfaction negatively despite the overall patient satisfaction was high [27]. A study conducted in a teaching hospital in Nigeria depicted that patient provider relationship, inpatient services, hospital facilities and access to care affected patient satisfaction positively whereas waiting time, cost, delayed appointment, missing investigation results and folders affected patient satisfaction negatively [28]. A comparative evaluation of patients’ satisfaction with cataract surgical services in a public tertiary and a private secondary eye care facilities in Nigeria found that patients were more satisfied with the pre-consultation time and cost of surgery [29]). A study done on determinants of patient satisfaction with outpatient health services at public and private hospitals in Addis Ababa, Ethiopia showed that self rated health status, expectation about the services, perceived adequacy of consultation duration, perceived providers’ technical competency, perceived welcoming approach and perceived body signalling were determinants of satisfaction from both public and private hospitals [30].

## Methods

### Study design and period

Hospital based cross-sectional study was conducted in University of Gondar teaching hospital from April1-30, 2014.

### Study area

University of Gondar hospital is one of the largest referral and teaching hospitals in Northwest Ethiopia that provides health services for about 5 million people. The hospital has about 500 beds and 17 wards. There are seven operation theatres; four for surgery and gyn-obs, one for ophthalmology and two for fistula. There is one recovery room for the recovery of surgery and gynaecology patients after operation. One obstetrics ward used as labouring ward and recovery of operated obstetrics patients. The hospital has medical and paediatrics ICU but no surgical ICU. Six thousand three hundred fifty operations were done under anaesthesia in 2013 (retrospective audit of operation registration books).

### Source and study population

**Source population**: All adult (minor-major, outpatient-inpatient, elective -emergency) surgery, obs-gyn (obstetrics, gynaecology, fistula) and ophthalmology patients who operated upon in University of Gondar teaching hospital.

**Study population**: All adult (minor-major, outpatient-inpatient, elective -emergency) surgery, obs-gyn (obstetrics, gynaecology, fistula) and ophthalmology patients who operated upon in University of Gondar teaching hospital during the study period were included in the study.

### Inclusion and exclusion criteria

**Inclusion criteria**: All adult (minor-major, outpatient-inpatient, elective -emergency) surgery, obs-gyn (obstetrics, gynaecology, fistula and ophthalmology who operated upon in University of Gondar teaching hospital during the study period were included.

**Exclusion criteria**: Patients who operated under local anaesthesia at outpatient department (OPD) level, patients below 18 years old, patients who cannot communicate, and unconscious after operation during data collection were excluded.

### Variables of the study

**Dependent variables**: Patient satisfaction (satisfactory and unsatisfactory)

### Independent variables

**Socio-demographic variables**: Age, sex, address (rural-urban), ethnicity, educational status, frequency of hospital visit ( new, repeated), reason for visit (illness,check-up,interview day waiting time),expectation of service (high,medium,low,none), self rating health status (very well,well,no change, getting worsen), frequency of operation (first, twice etc),duration of hospital stay, ASA status, presence of co-morbidity, type of operation (minor-major, elective-emergency, outpatient-inpatient, and surgery, obs-gyn and ophthalmology) and duration of surgery (short-long).

**Factors related with reception at OPD, preoperative visit and preparation**: Nurses/physician concern for patient’s problem during presentation, concern for privacy during examination, adequacy of the answer for patients’ questions, adequacy of information about the nature of the problem or operation, adequacy of information regarding possible complications and their management after operation, cleanliness of the site for the patient, chance to know the importance of the investigations ordered and waiting time.

**Factors related with reception in the operation theatre and operation**: Taking in to account privacy, open attitude, being respectful, understanding patient’s situation, staff politeness, being professional, paying attention to patient’s question, attention to complaints like pain, taking in to account patient’s preference, staff knowledgeable and treating kindly.

**Factors related with the postoperative management in the wards**: nNurses maintain privacy, skill, sympathy for patients, prompt response for patient call, respect during communication, information about patient health status or progress, clear information about the investigations, information about side effect of medications, spend adequate time with the patient during treatment and adequate care at night. And medical staff’s (physicians): responsible for you, communicate in understandable way, available when needed, show a carrying attitude, confidence in the skill of physicians. And hospital facility: cleaning of wards/beds, cleaning of bathing room, cleaning of toilet, adequacy of food and water, accessibility of pharmacy and laboratory and cost for medications and investigations.

**Factors related with the postoperative complication and discomforts**: Pain, PONV, sore throat, depression, discomfort, hunger, thirst, bleeding and re-operation including death.

### Operational definitions

**Surgical patients**: Mean all adult minor-major, outpatient-inpatient, elective -emergency surgery and obs-gyn (obstetrics, gynaecology, fistula)y patients who operated during the study period.

**Satisfaction**: Attaining one’s need or desire.

**Very satisfactory**: Above one’s expectation.

**Satisfactory**: Just one’s expectation.

**Dissatisfactory**: Below one’s expectation.

**Very dissatisfactory**: Fail to meet one’s expectation usually leading to disappointment.

**Patient waiting time**: The interval between departure from the proceeding outpatient station and receiving service at the next outpatient station

### Sample size and sampling procedure

All consecutive adult (minor-major, outpatient-inpatient, elective-emergency) surgery, obs-gyn (obstetrics, gynaecology, fistula) and ophthalmology patients who operated at the University of Gondar teaching hospital during the study period were included.

### Data collection procedures

A pre-tested, structured and Amharic version questionnaire used to interview patients within 24 hours after operation after the patients are fully awake. The Amharic version questionnaire was pre-tested before actual data collection in an area not included in the research area. Checklist was used to extract data from the patients’ chart and anaesthetic record sheets. Two BSc holder data collectors were selected and one day training was given.

### Data quality control

To ensure the quality of data, training was provided for data collectors and the investigators directed and monitored the whole data collection processes for consistency, completeness and accuracy. Pre-test was done, data cleaned and checked every day, and double data entry technique used during data entry.

### Data processing and analysis

The data coded, entered and analyzed using SPSS version 20 software. Associations between dependent and independent variables were assessed and its strength was presented using adjusted odds ratios and 95% confidence interval. Binary and multiple logistic regressions used to assess the association between outcome and explanatory variables. Variables from the bivariate analysis fitted for the outcome variables in relation to each explanatory variable. Those variables which fulfilled the minimum requirement of 0.2 level of significance were further entered in to multivariate logistic analysis for further assessment and the fitness of the model was checked using Hosmer and Lemeshow goodness of fitness. Frequency tables and summary statistics were used.

### Ethical considerations

Ethical clearance obtained from the institutional ethical review board of University of Gondar. Official permission letter obtained from University of Gondar hospital. Oral informed consent was obtained from each study subject after explanation of what they will take part in the research and any involvement was after their complete consent. Anyone not willing to participate in the study have had full right not to participate. Confidentiality was ensured from all the data collectors and investigators by avoiding personal identification on the questionnaire and keeping questionnaires locked.

## Results

### Socio-demographic characteristics of the study participants

Two hundred and seventy eight patients underwent surgery during the study period. Nine patients were excluded due to refusal to participate in the study. A total of 269 out of 278 patients were included in the study with a response rate of 96.8%. One hundred and fifty eight out of 269 (58.7%) patients were males. The minimum, maximum and median age of the patients were 18,82 and 30+12.98 years respectively. The age of 132 patients was in the range of 18-29 years, 105 patients in the range of 30-49 years, 24 patients in the range of 50-65 years and 8 patients >65 years old respectively. The majority of patients (n=168, 62.5%) came from the rural area whereas 101 (37.5%) from urban area. One hundred and seven patients were illiterate (39.8%), only write and read 41(15.2%), primary education 46 (17.1%), secondary education 42 (15.6%), certificate and diploma 11 (4.1%), and degree and above 22 (8.2%) respectively. Two hundred and thirty two patients were Amhara (86.2%), Tigray 36 (13.4%) and Oromo 1 (0.4%). The majority of patients were American Society of Anesthesiologist's class one: ASA1 (n=231, 85.9%), ASA2 (n=33, 12.3%) and ASA3 (n=5, 1.9%). Two hundred and twenty one (82.2%) patients have had major operation whereas the rest underwent minor operation (n=48, 17.8%). The majority of operations were emergency (n=174, 64.7%) and 95 (35.3%) were elective operations. Most operations were surgery (n=238, 88.5%) whereas 31 (11.5%) were obs-gyn operation. Two hundred and three patients were interviewed at recovery room (n=203, 75.5%), gynaecology ward 11(4.1%), obstetrics ward 20 (7.4%), general ward 2 (0.7%), surgical ward 13 (4.8%), orthopaedic ward 6 (2.2%) and trauma unit 14 (5.2%) respectively. The minimum, maximum and median duration of surgery were 30, 360 and 70+49.18 minutes respectively. The minimum, maximum and median length of hospital stay were 8 hours, 5328 hours and 55+375.59 hours respectively (Table 1).

**Table 1:** Socio-demographic variables

Variable	Frequency	Percentage (%)
**Frequency of hospital visit**		
First	210	78.1
Second	59	21.9
Reason for hospital visit		
Illness	258	95.9
Check up	9	3.3
Interview	2	0.7
**Patient's service expectation**		
High	192	71.4
Medium	56	20.8
Low	12	4.5
None	9	3.3
**Frequency of operation**		
First	245	91.1
Repeated	24	8.9
**Payment status for treatment**		
Paid	264	98.1
Free	5	1.9
**Self-rated health status during interview**		
Very well	135	50.2
Well	123	45.7
No change	11	4.1

### Patient satisfaction with outpatient department (OPD) services

Patients' satisfaction with physicians’ and nurses’ concern for their problem at presentation at the outpatient department were very satisfied 92 (34.2%), satisfied 164 (61%), neutral 2 (0.7%), dissatisfied 9 (3.3%) and very dissatisfied (0.7%). Patients' satisfaction with the physicians' and nurses' concern for their privacy during examination at outpatient department were very satisfied 91 (33.8%), satisfied 167 (62.1%), neutral 1(0.4%), dissatisfied 7(2.6%) and very dissatisfied 3 (1.1%) (Table 2).

**Table 2:** Patient satisfaction with OPD services

Variable	Very Satisfied n (%)	Satisfied n (%)	Neutral n (%)	Dissatisfied n (%)	Very Dissatisfied n (%)
Adequacy of physicians’ and nurses’ answer for your questions?	90 (33.5)	167 (62.1)	2 (0.7)	10 (3.7)	0 (0)
Adequacy of physicians’ and nurses’ information about the nature of your problem or operation?	91 (33.8)	164 (61)	2 (0.7)	11 (4.1)	1 (0.4)
Adequacy of information provided about the possible complications that may occur during operation or treatment?	91 (33.8)	162 (60.2)	6 (2.2)	9 (3.3)	1 (0.4)
Adequacy of information about the importance of the investigations?	70 (26)	161 (59.9)	26 (9.7)	9 (3.3)	3 (1.1)
Waiting time to see the doctor?	56 (20.8)	163 (60.6)	12 (4.5)	29 (10.8)	3 (1.1)
Waiting time to give laboratory specimens?	42 (15.6)	156 (58)	51 (19)	14 (5.2)	6 (2.2)
Waiting time to see the doctor after receiving lab results	51 (19)	142 (52.8)	51 (19)	21 (7.8)	4 (1.5)
How did you get the cleanliness of the outpatient ward or clinic?	53 (19.7)	194 (72.1)	9 (3.3)	10 (3.7)	3 (1.1)

### Patient satisfaction with operation theatre services

Patient satisfaction with operation theatre staff consideration about patient privacy were very satisfied 75 (27.9%), satisfied 163 (60.6%), neutral 17 (6.3%), dissatisfied 13 (4.8%) and very dissatisfied 1(0.4%). Patient satisfaction with operation theatre staff open attitude for patients were very satisfied 74 (27.5%), satisfied 171 (63.6%), neutral 16 (5.9%), dissatisfied 8 (3%) and very dissatisfied 0 (0%) (Table 3).

**Table 3:** Patient satisfaction with operation theater services

Variable	Very Satisfied n (%)	Satisfied n (%)	Neutral n (%)	Dissatisfied n (%)	Very Dissatisfied n (%)
Respective fullness of operation theatre staff for you?	94 (34.9)	150(55.8)	16(5.9)	8 (3)	1 (0.4)
Did the operation theatre staff understand your situation?	93 (34.6)	154 (57.2)	15 (5.6)	6 (2.2)	1 (0.4)
Politeness of the operation theatre staffs for you?	106 (39.4)	140 (52)	12 (4.5)	10 (3.7)	1 (0.4)
The professionalism of the operation theatre staffs?	65 (24.2)	175 (65.1)	24 (8.9)	4 (1.5)	1 (0.4)
The attention of the operation theatre staff to your questions?	59 (21.9)	182 (67.7)	17 (6.3)	10 (3.7)	1 (0.4)
Did the operation theatre staff give attention to your complaint like pain or nausea?	55 (20.4)	175 (65.1)	24 (8.9)	13 (4.8)	2 (0.7)
Did the operation theatre staff take in to account your preference?	57 (21.2)	183 (68)	10 (3.7)	18 (6.7)	1 (0.4)
Did the operation theatre staff treat you kindly?	60 (22.3)	192 (71.4)	11 (4.1)	4 (1.5)	2 (0.7)
Were you confident that the operation theatre staffs were knowledgeable and skilful?	51 (19)	186 (69.1)	30 (11.2)	1 (0.4)	1 (0.4)

### Patient satisfaction with postoperative management in the wards

Patient satisfaction with health provider maintenance of patients' privacy was very satisfied 118 (43.9%), satisfied 143 (53.2%), neutral 2 (0.7%), dissatisfied 5 (1.9%) and very dissatisfied 1(0.4%). Patient satisfaction with ward nurse skill of treatment was very satisfied 110 (40.9%), satisfied 137 (50.9%), neutral 20 (7.4%), dissatisfied 2 (0.7%) and very dissatisfied 0 (%). Patient satisfaction with ward nurse sympathy was very satisfied 122 (45.4%), satisfied 136 (50.6%), neutral 4 (1.5%), dissatisfied 7 (2.6%) and very dissatisfied 0(0) (Table 4, Table 5).

**Table 4:** Patient satisfaction with postoperative management in the wards

Variable	Very Satisfied n (%)	Satisfied n (%)	Neutral n (%)	Dissatisfied n (%)	Very Dissatisfied n (%)
Nurses’ provide prompt response for your call?	127 (47.2)	137 (50.9)	4 (1.%)	1 (0.4)	0(0)
Ward nurses respection to you during communication?	144 (53.5)	116 (43.1)	6 (2.2)	2 (0.7)	1 (0.4)
Adequacy of ward nurses’ information to you about your health progress?	106 (39.4)	134 (49.8)	19 (7.1)	9 (3.3)	1 (0.4)
Ward nurses’ information about the importance of investigations?	77 (28.6)	136 (50.6)	33 (12.3)	21 (7.8)	2 (0.7)
Adequacy of information provided by ward nurses about the side effects of medications?	52 (19.3)	105 (39)	55 (20.4)	56 (20.8)	1 (0.4)
Adequacy of time the ward nurses spent with you during evaluation and treatment?	77 (28.6)	166 (61.7)	16 (5.9)	9 (3.3)	1 (0.4)
Adequacy of the care given by ward nurses at night?	71 (26.4)	177 (65.8)	17 (6.3)	4 (1.5)	0 (0)
Responsibility of the physician for you?	85 (31.6)	176 (65.4)	4 (1.5)	3 (1.1)	1 (0.4)
The communication of the physicians with you in an understandable way?	109 (40.5)	150 (55.8)	6 (2.2)	4 (1.5)	0 (0)

**Table 5:** Patient satisfaction with postoperative management in the wards (continued)

Variable	Very Satisfied n (%)	Satisfied n (%)	Neutral n (%)	Dissatisfied n (%)	Very Dissatisfied n (%)
The availability of the responsible physician when needed?	69 (25.7)	184 (68.4)	6 (2.2)	9 (3.3)	1 (0.4)
Did the physician show a carrying attitude for you?	72 (26.8)	185 (68.8)	5 (1.9)	7 (2.6)	0 (0)
Were you confident in the skill of physicians during treatment?	66 (24.5)	143 (53.2)	55 (20.4)	5 (1.9)	0 (0)
The cleanliness of the ward or beds?	58 (21.6)	177 (65.8)	12 (4.5)	18 (6.7)	4 (1.5)
The cleanliness of the bath room?	43 (16)	149 (55.4)	40 (14.9)	29 (10.8)	8 (3)
The cleanliness of the latrines?	44 (16.4)	149 (55.4)	40 (14.9)	25 (9.3)	11 (4.1)
The adequacy of food and water supply?	35 (13)	120 (44.6)	57 (21.2)	43 (16)	14 (5.2)
Accessibility of pharmacy and laboratory facilities to you?	34 (12.6)	86 (32)	33 (12.3)	76 (28.3)	40 (14.9)
The fairness of medication and investigation costs for you?	29 (10.8)	154 (57.2)	28 (10.4)	36 (13.4)	22 (8.2)

### Patient satisfaction with information provision about postoperative complications and treatment options

Patient satisfaction with information provision about the risk of postoperative pain and treatment options was very satisfied 54 (20.1%), satisfied 138 (51.3%), neutral 72 (26.8%), dissatisfied 5 (1.9%) and very dissatisfied 0 (0) (Table 6).

**Table 6:** Patient satisfaction with information provision about postoperative complications and treatment options

Variable	Very Satisfied n (%)	Satisfied n (%)	Neutral n (%)	Dissatisfied n (%)	Very Dissatisfied n (%)
Information provided by health professionals about the risk of PONV and treatment options?	49 (18.2)	128 (47.6)	87 (32.3)	5 (1.9)	0 (0)
Information provided by health professionals about the risk of sore throat and treatment options after operation?	43 (16)	99 (36.8)	121 (45)	5 (1.9)	1 (0.4)
Information provided by health professionals about the risk of depression and treatment options after operation?	39 (14.5)	82 (30.5)	136 (50.6)	12 (4.5)	0 (0)
Information provided by health professionals about the risk of discomfort and relieving methods after operation?	39 (14.5)	84 (31.2)	139 (51.7)	7 (2.6)	0 (0)
Information provided by health professionals about the risks of hunger and thirst and treatment options after operation?	41 (15.2)	91 (33.8)	131 (48.7)	6 (2.2)	0 (0)
Information provided by health professionals about the risk of bleeding and treatment options after operation?	49 (18.2)	122 (45.4)	92 (34.2)	5 (1.9)	1 (0.4)
Information provided by health professionals about the risks of re-operation and death after operation?					

### Overall patient satisfaction level with perioperative surgical services and associated factors

The overall level of patient satisfaction with perioperative surgical services at the University of Gondar Teaching and Referral hospital was 98.1%. The variables that had p-value of < 0.2 from the bivariate analysis but had no association from the multivariate analysis were physician and nurse concern for the patients' problem, physician and nurse maintaining the patients' privacy, adequacy of answer of care providers for the patients' questions, adequacy of information about the risk of postoperative complications and treatment options, information about the health progress of the patients in the ward, information about the importance of investigations, information about the side effects of medications, the responsibility of the physician for the patients, physician communication in understandable way and the fairness of medication and investigation costs. The variables that had association with the outcome variable from the multivariate analysis were patient admission status (AOR=0.073, CI=0.007-0.765, P=0.029), information about the disease and operation (AOR=0.010, CI=0.001-0.140, P=0.001) and operation theatre staff attention to the patients complains (AOR=0.028, CI=0.002-0.390, P=0.008) respectively.

## Discussion

This study revealed that the level of patient satisfaction with the perioperative surgical services was 98.1%. This finding was high compared with the previous studies conducted in our country [2, 3, 30] and in other countries in the world [23, 26]. This discrepancy could be due to the difference in patient perception of the services rendered and study design. In this study, from the outpatient department the adequacy of physicians’ and nurses’ information about the nature of your problem or operation cause dissatisfaction 11 (4.1%), waiting time to see the doctor dissatisfied 29 (10.8%), waiting time to give laboratory specimens dissatisfied 14 (5.2%) and waiting time to see the doctor after receiving lab results dissatisfied 21 (7.8%) patients respectively. These degrees of dissatisfactions were low compared with a previous study conducted in Jimma University Specialized hospital [2] which might be attributed to relatively low number of patient flow to the outpatient department in our hospital. From the operation theatre, respective fullness of operation theatre staff for you dissatisfied 8 (3%), politeness of the operation theatre staffs for patients dissatisfied 10 (3.7%), the attention of the operation theatre staff to the patients' questions dissatisfied 10 (3.7), the operation theatre staff give attention to the patients' complaint like pain or nausea dissatisfied 13 (4.8%) and the operation theatre staff take in to account the patients' preferences dissatisfied 18 (6.7%) patients respectively. This could be due to the operation theatre staffs might be very busy with different activities which reduce the attention for patients complaints. In the present study, the major areas of patient dissatisfaction in the postoperative patient management in the wards were; ward nurses’ information about the importance of investigations 21 (7.8%), the adequacy of information provided by ward nurses about the side effects of medications dissatisfied 56 (20.8%), the cleanliness of the ward or beds 18 (6.7%), the cleanliness of the bath room 29 (10.8%), the cleanliness of the latrines 25 (9.3%), the adequacy of food and water supply 43 (16%), accessibility of pharmacy and laboratory facilities to you 76 (28.3%) and the fairness of medication and investigation costs for you 36 (13.4%). These findings were low compared with previous studies [2, 3, 30] that might be due relative low patients flow rate and a difference in patient characteristics that attributed to perceived satisfaction variation. In this study, information provision on the risk of postoperative different complications and treatment options caused patient dissatisfaction in the areas of information provided by health professionals about the risk of depression and treatment options after operation 12 (4.5%), information provided by health professionals about the risk of discomfort and relieving methods after operation 7 (2.6%), and information provided by health professionals about the risks of hunger and thirst and treatment options after operation 6 (2.2%). Information provision before and after treatments are crucial for patient satisfaction with health services rendered [20].

## Conclusion

This study revealed that the level of patient satisfaction with the perioperative surgical services was high compared with previous studies conducted in the country and other countries in the world. Patient admission status, information about the disease and operation status and operation theatre staff attention to the patients complaints were the determinant factors for patient satisfaction. Health professionals need to give emphasis for information on care provision processes, patients' health progress and patients' complaints.

### What is known about this topic

- Patient satisfaction with surgical services is an indicator of quality improvement;
- Patient satisfaction can be affected by different factors.

### What this study adds

- Revealed as some areas of services need special attention particularly in resource limited settings;
- Enable to prioritize the services as the gaps are identified;
- Helps to develop service improving strategies based on the gaps.

## Competing interests

The authors declare no competing interest.
